# Storage of Lithium-Ion by Phase Engineered MoO_3_ Homojunctions

**DOI:** 10.3390/nano12213762

**Published:** 2022-10-26

**Authors:** Dickon H. L. Ng, Sheng Li, Jun Li, Jinning Huang, Yingxue Cui, Jiabiao Lian, Chuan Wang

**Affiliations:** 1 School of Science and Engineering, The Chinese University of Hong Kong (Shenzhen), Longgang, Shenzhen 518172, China; 2 Key Laboratory of Zhenjiang, Institute for Energy Research, Jiangsu University, Zhenjiang 212013, China; 3 Institute of Advanced Synthesis, School of Chemistry and Molecular Engineering, Jiangsu National Synergetic Innovation Center for Advanced Materials, Nanjing Tech University, 30 Puzhu South Road, Nanjing 211800, China

**Keywords:** anode materials, phase engineering, molybdenum trioxide, homojunctions, lithium-ion storage

## Abstract

With high theoretical specific capacity, the low-cost MoO_3_ is known to be a promising anode for lithium-ion batteries. However, low electronic conductivity and sluggish reaction kinetics have limited its ability for lithium ion storage. To improve this, the phase engineering approach is used to fabricate orthorhombic/monoclinic MoO_3_ (α/h-MoO_3_) homojunctions. The α/h-MoO_3_ is found to have excessive hetero-phase interface. This not only creates more active sites in the MoO_3_ for Li^+^ storage, it regulates local coordination environment and electronic structure, thus inducing a built-in electric field for boosting electron/ion transport. In using α/h-MoO_3_, higher capacity (1094 mAh g^−1^ at 0.1 A g^−1^) and rate performance (406 mAh g^−1^ at 5.0 A g^−1^) are obtained than when using only the single phase h-MoO_3_ or α-MoO_3_. This work provides an option to use α/h-MoO_3_ hetero-phase homojunction in LIBs.

## 1. Introduction

Owing to the high energy density, flexibility in design and long cyclic life, lithium-ion batteries (LIBs) are playing essential roles in our modern society, from domestic electronic products to electric vehicles (EVs) and aerospace carriers [1,2]. Nonetheless, current commercial graphite electrodes provide limited rate capacity and having sluggish Li^+^ intercalation which are insufficient to meet the demands of the next generation of LIBs [3]. It is highly desirable to exploit new anode materials with higher rates to meet demands for various applications [4,5]. Among the commonly used transition metal oxides for anode materials, the low cost MoO_3_ has relatively high theoretical specific capacity (1117 mA h g^−1^, much higher than commercial graphite (372 mA h g^−1^)) [6,7,8,9]. However, the Li^+^ storage performance of MoO_3_ is suppressed by the inherently low electronic conductivity, sluggish Li^+^ diffusion kinetics and the large volume change, resulting in severe polarization and poor rate performance [10,11]. In the exploration aiming to enhance the kinetics of MoO_3_, some of the work involved the designing of nano/micro-architecture to increase the contact area with the electrolyte [12,13,14], incorporating a carbon-based conductive matrix to enhance the stability [15,16,17], and introducing defects to optimize conductivity [18,19]. To some extent, the above-mentioned strategies have improved structure stability and electrical conductivity, while the intrinsic slow ion migration property of MoO_3_ remains and impedes the utilization of MoO_3_ [20,21,22]. Thus, it is imperative to exploit universal strategies to boost the electrochemical performance of the MoO_3_. Fortunately, phase engineering is an effective and powerful strategy to modulate the physicochemical properties and functionalities by tailoring transformation of different phases in nanomaterials [23,24,25,26,27,28]. In previous research, Song et al. studied cubic/orthorhombic-CoSe_2_ homojunctions, and found that these heterojunctions would redistribute the interfacial charges and accelerated the ion diffusion, resulting in better performance of the sodium/potassium storage than those using cubic or orthorhombic CoSe_2_ [29]. It is evident that the different crystallographic structure determines its unique electrochemical property. The MoO_3_ usually exists in two structural phases, namely orthorhombic (α-MoO_3_), and monoclinic (h-MoO_3_). It is highly feasible to make and use hetero-phase homojunctions for MoO_3_ as electrode materials for electrochemical performance.

In this work, orthorhombic/monoclinic MoO_3_ (α/h-MoO_3_) homojunctions were fabricated via partial phase transformation with a facile heat treatment. Compared with heterojunctions, the homojunction with interfaces exhibit stronger electron coupling due to their similar physicochemical properties and lattice matching. At the unique homojunction interfaces, the unbalanced charge distribution would also create an internal electric field, which facilitates the interfacial charge transport, accelerates ion diffusion, increases the reactive sites and improves the electrochemical surface reaction kinetics. The work demonstrates that α/h-MoO_3_ electrodes could delivered an extraordinarily high capacity of 1094 mAh g^−1^ at 100 mA g^−1^ with outstanding cyclic stability and a capacity of 462 mAh g^−1^ remaining after 300 cycles of operation at 1.0 A g^−1^. This work confirms that, via constructing the hetero-phase homojunction in MoO_3_, Li^+^, storage kinetics can be largely enhanced.

## 2. Materials and Methods

### 2.1. Material Preparation

The monoclinic (h-MoO_3_), orthorhombic/monoclinic MoO_3_ (α/h-MoO_3_) and orthorhombic MoO_3_ (α-MoO_3_) samples were prepared as follows. For synthesis of a typical sample, 2.0 g of Ammonium molybdate tetrahydrate ((NH_4_)_6_Mo_7_O_24_·4H_2_O) was dispersed into 10 mL deionized water before 1 mL HNO_3_ was added into the solution under stirring for 30 min. The mixture was transferred to a 50 mL autoclave and heated at 180 °C for 12 h. The product was collected and washed with deionized water and ethanol before being dried in an oven at 60 °C overnight. This product was named Sample A. Subsequently, Sample A was heat-treated in a tube furnace at 400 °C for 30 min to produce Sample B. Thereafter, Sample B was further treated at 450 °C for 30 min to obtain Sample C.

### 2.2. Characterization

The composition and phase information of the samples were determined by X-ray diffractometry (XRD, Bruker D8, Mannheim, Germany). The morphology and microstructure of the samples were examined by the scanning electron microscope (SEM, JEOL, JSM-7800F, Tokyo, Japan). The Raman spectra were performed by using a DXR Raman system spectrometer (Thermo Scientific, Waltham, MA, USA) with a 532 nm laser.

### 2.3. Electrochemical Measurements

Cyclic voltammetry (CV) and electrochemical impedance spectroscopy (EIS) measurements were conducted on a Gamry (Interface 1000 E Potentiostat) electrochemical workstation. The galvanostatic discharge and charge profiles were measured by the NEWARE-CT-4008 battery tester. The working electrodes were prepared by mixing the MoO_3_, super conductive carbon black (SCCB, Ketjenblack EC-600JD, Lion Corporation, Tokyo, Japan) and polyvinylidene fluorid (PVDF) binder in N-methyl-2-pyrrolidone (NMP) with a weight ratio of 8:1:1. After stirring for 1 h, the homogeneous slurry was coated onto a copper foil. The mass loading of the active material was estimated to be about 1.0 mg cm^−2^. The CR2032 coin-type cells using the fabricated product were assembled in an argon-filled glovebox (oxygen/moisture concentrations < 0.01 ppm). Celgard 2400 porous polypropylene membrane was used as the separator. The electrolyte is 1 M LiPF_6_ in carbonate (EC)/dimethyl carbonate (DMC) with the volume ratio of 1:1.

## 3. Results and Discussion

**Figure 1:**
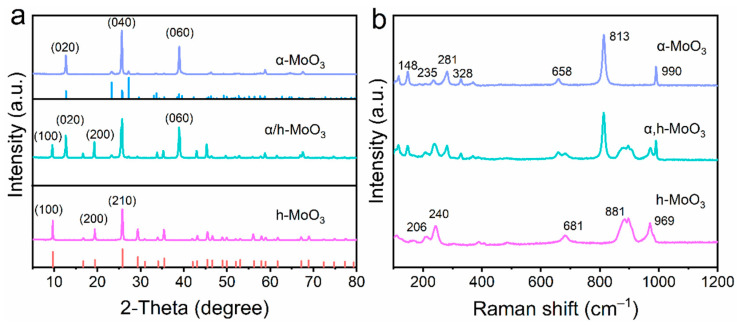
(**a**) XRD patterns and (**b**) Raman spectra of Sample A: h-MoO_3_, Sample B: α/h-MoO_3_, and Sample C: α-MoO_3_.

Powder X-ray diffractometry (XRD) and Raman scattering (RS) spectroscopy were used to investigate the phase structures and purity of MoO_3_ with different phases. As shown in Figure 1a, the narrow and sharp diffraction peaks in the XRD patterns indicate the superior crystallinity of h-MoO_3_ (Sample A), α/h-MoO_3_ (Sample B), and α-MoO_3_ (Sample C). The characteristic peaks at 9.6°, 19.4°, 25.7°, 29.3°, 35.4°, 45.5°, and 69° can be assigned to (100), (200), (210), (300), (310), (410), and (524) of hexagonal MoO_3_ (PDF#21-0569) [27,30]. No other peaks are observed in the pattern of the h-MoO_3_ (top of Figure 1a), indicating that there are no impurities. The middle pattern of α/h-MoO_3_ in Figure 1a contains new peaks at 12.8°, 23.2°, 25.5°, and 27.1°, which can be indexed to the (020), (110), (040), and (021) planes of α-MoO_3_, respectively (PDF#35-0609) [15]. It is evident that h-MoO_3_ under air atmosphere partially transfers to α/h-MoO_3_ at 400 °C. The subsequent heat treatment of α/h-MoO_3_ leads to the formation of thermodynamically stable α-MoO_3_ pillar at 450 °C as confirmed in the bottom pattern in Figure 1a. To better elucidate the crystal structures, Raman characterization was performed and the results are shown in Figure 1b. The signal peaks at 206 cm^−1^, 240 cm^−1^, 681 cm^−1^, 881 cm^−1^, and 969 cm^−1^ are attributed to h-MoO_3_ [24]. Meanwhile, the Raman spectrum of the α-MoO_3_ shows the characteristic bands at 148 cm^−1^, 235 cm^−1^, 281 cm^−1^, 328 cm^−1^, 658 cm^−1^, 813 cm^−1^, and 990 cm^−1^ [15]. Notably, the Raman peaks correspond to α/h-MoO_3_ indicating that the hexagonal and orthorhombic crystal phases coexist, which is consistent with the XRD results (Figure 1a). The characterization results suggest the successful generation of homojunctions in α/h-MoO_3_, which is expected to synergize the benefits from different phases for improving the electrochemical performance.

**Figure 2:**
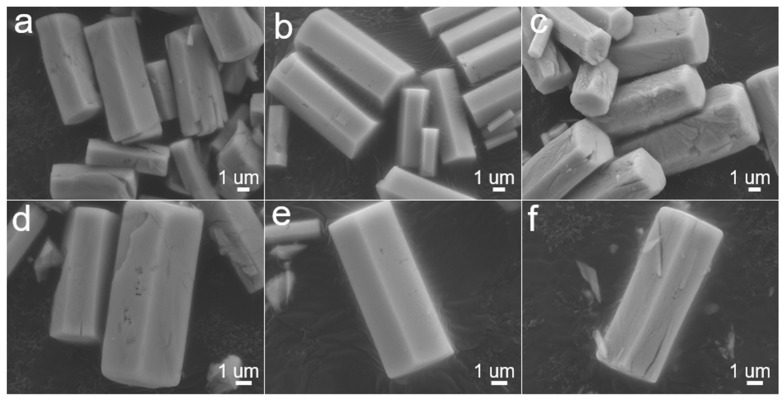
SEM images of (**a**,**d**) Sample B: α/h-MoO_3_, (**b**,**e**) Sample A: h-MoO_3_, and (**c**,**f**) Sample C: α-MoO_3_, respectively.

The morphological characteristics of the MoO_3_ samples were investigated by SEM. The homogeneous hexagonal crystals were clearly observed and shown in Figure 2. Figure 2a–c all show similar hexagonal prism morphology with a length of about 10 μm, indicating their morphology was preserved during the heat treatment. In the high-magnification images (Figure 2d–f), an interesting conversion was observed. Compared to h-MoO_3_, the surface of α/h-MoO_3_ and α-MoO_3_ appears rough, which is attributed to the evolution of gases during heat treatment.

**Figure 3:**
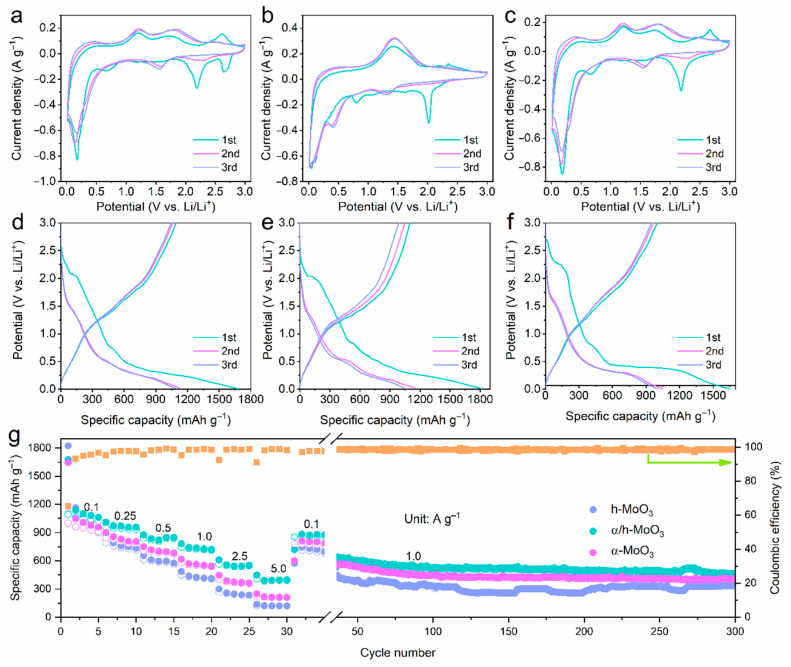
Electrochemical Li^+^ storage performance of MoO_3_ electrodes. The initial three CV curves at 0.1 mV s^−1^ and GCD profiles at 0.1 A g^−1^ of (**a**,**d**) Sample B: α/h-MoO_3_; (**b**,**e**) Sample A: h-MoO_3_; (**c**,**f**) Sample C: α-MoO_3_. (**g**) Rate capability and long-term cycling stability.

The Li^+^ storage behavior of MoO_3_ electrodes were systematically evaluated in a coin cell configuration in a potential window of 0.01 V to 3.0 V. Figure 3 shows the results of the electrochemical processes of α/h-MoO_3_, h-MoO_3_ and α-MoO_3_. Their initial three cyclic voltammetry (CV) curves at a scan rate of 0.1 mV s^−1^ are shown in Figure 3a–c, while their corresponding initial three GCD profiles are displayed in Figure 3d–f. It is noted that, in the first cathodic sweep, the irreversible reduction peak occurring at 2.6 V and 2.2 V could be assigned to the multistep Li^+^ insertion into MoO_3_ (MoO_3_ + xLi^+^ + xe^−^ ↔  Li_x_MoO_3_); the irreversible peak at 0.7 V is due to the decomposition of electrolytes and the formation of the SEI layer, which disappears in the following cycles and thus causes part of the observed irreversible capacity during the first cycle [17]. On the other hand, a reduction peak at 0.17 V corresponds to the formation of Mo metal by conversion reaction (Li*_x_*MoO_3_ + (6 − *x*) Li^+^ + (6 − *x*) e^−^↔ Mo + Li_2_O) [19]. 

In the anodic scans, the observed two oxidation peaks around 1.2 V, 1.7 V, and 2.6 V were attributed to the reversible reaction from Mo and Li_2_O to Li*_x_*MoO_3_ during delithiation process, respectively [31]. The second and third cathodic scans show the lithium intercalation at 1.6 V and the conversion reaction peak at 0.13 V, indicating an amorphous structure of α/h-MoO_3_ is formed [15]. Meanwhile, the two CV curves almost overlapping suggests the good reversibility of the α/h-MoO_3_ electrode during the repeated Li^+^ insertion and extraction. Compared to the CV profiles of h-MoO_3_ (Figure 3b), there are some differences, e.g., that the conversion reaction occurs at 0.37 V and one broad anodic peak at 1.4 V corresponds to the reversible Li^+^ extraction. The CV evolution of α-MoO_3_ (Figure 3c) displays similar features to α/h-MoO_3_. Moreover, to highlight the different electrochemical behavior of these three electrodes, their first CV cycles were integrated in one image, as shown in Figure S1. Impressively, the initial three galvanostatic discharge-charge (GCD) profiles of the α/h-MoO_3_, h-MoO_3_, and α-MoO_3_ electrodes at current density of 0.1 A g^−1^ is shown in Figure 3d–f, respectively, where the platforms agree well with the CV results (Figure 3a–c). The initial discharge and charge capacities were 1677 and 1091 mAh g^−1^, with an initial Coulombic efficiency of 65%. It is higher than h-MoO_3_ and α-MoO_3_ (Figure 3e,f). The low initial coulombic efficiency might be caused by irreversible processes of SEI film and the capture of Li^+^ in the MoO_3_ lattice, which is also observed in initial CV cycles [32]. In successive charge–discharge cycles, it should be noticed that the GCD profiles of α/h-MoO_3_ become stable with a coulombic efficiency of 95%, but the gradual capacity fade of h-MoO_3_ and α-MoO_3_ is obvious. This suggests a good Li^+^ storage reversibility of the α/h-MoO_3_ electrode, due to the orthorhombic/monoclinic homojunctions facilitating the reversible lithiation/delithiation process by increasing the electronic conductivity and mechanical integrity of the MoO_3_ electrode against structural changes. The rate and cycle performance comparison are presented in Figure 3g. The α/h-MoO_3_ electrode displays reversible specific capacities 1094, 974, 846, 742, 562, and 406 mAh g^−1^ with current densities increased from 0.1 to 0.25, 0.5, 1.0, 2.5, and 5.0 A g^−1^, respectively. As the current density regularly returns to 0.1 A g^−1^, the reversible capacity of 846 mAh g^−1^ can still be obtained, suggesting good electrochemical reversibility. On the contrary, the h-MoO_3_ and α-MoO_3_ electrodes show a poorer rate of capability, resulting in lower capacities (125 and 222 mAh g^−1^) at high current density 5.0 A g^−1^. Figure S2 shows the corresponding galvanostatic charge–discharge (GCD) profiles for the α/h-MoO_3_, h-MoO_3_ and α-MoO_3_ electrodes at various current rates. It is obvious that the charge/discharge profiles of the α/h-MoO_3_ electrode displays much lower polarization at varied current densities [32], illustrating better reversible performance and less polarization at a high current. Therefore, it can be inferred that the homogenous orthorhombic/monoclinic interface with more Li storage active sites and enhanced electronic conductivity and Li^+^ transport [33,34], resulting in high capacities and superior rate performance for the α/h-MoO_3_ electrode. Subsequently, the long-term cycling stabilities of different electrodes was examined at 1.0 A g^−1^. It is obvious that the α/h-MoO_3_ electrode delivered a reversible capacity of 462 mAh g^−1^ over 300 cycles and satisfying capacity retention of 74%, which is higher than those of h-MoO_3_ (339 mAh g^−1^) and α-MoO_3_ (409 mAh g^−1^). The above results evidentially demonstrate that the homojunctions effectively alleviate the pulverization and minimize the volume change to maintain structural integrity during the Li^+^ insertion/extraction process [21].

**Figure 4:**
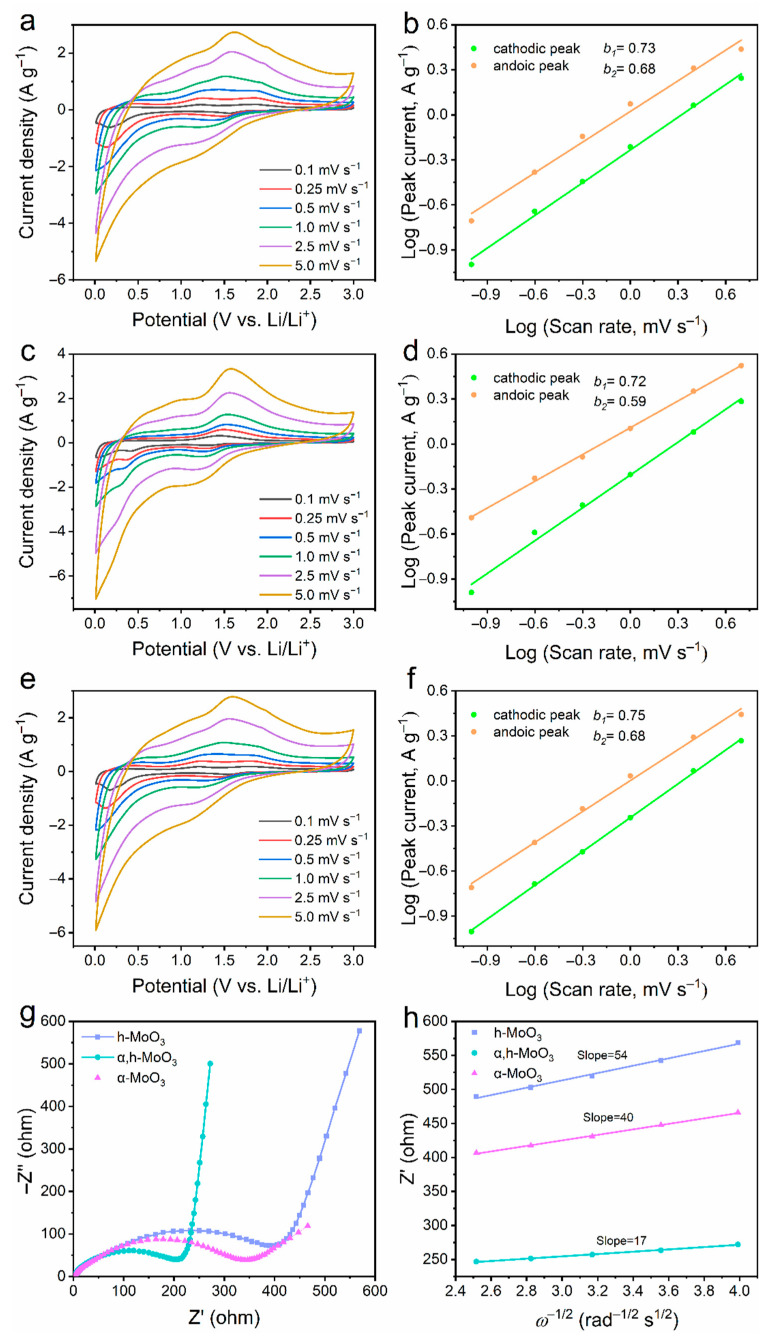
The CV curves at different scan rates and corresponding *b*-value determinations of (**a**,**b**) Sample B: α/h-MoO_3_, (**c**,**d**) Sample A: h-MoO_3_, and (**e**,**f**) Sample C: α-MoO_3_. (**g**) Electrochemical impedance spectra and (**h**) *Z*′−*ω*^−1/2^ plots.

In order to have better understanding of the role of the homojunction structure for such enhanced rate capability of α/h-MoO_3_ electrodes, the CV curves at various scan rates of 0.1–5.0 mV s^−1^ (Figure 4a,c,e) were conducted to investigate Li^+^ storage kinetics. In principle, the relationship between current i and scan rate *v* was investigated according to the following power law [35]:

*i* = *av^b^* (1)

*log(i)* = *blog(v)* + *log(a)* (2)

where i is peak current, v is sweep rate, a and b are adjustable values. When the *b*-Value closes to 0.5 indicating diffusion-controlled mechanism or 1.0 corresponding to capacitive-controlled process for Li^+^ storage behaviors, respectively. The corresponding fitted line for both cathodic and anodic peaks currents can be found in Figure 4b,d,f. The *b* value of α/h-MoO_3_, h-MoO_3_ and α/h-MoO_3_ could be obtained as 0.73/0.68, 0.72/0.59, and 0.75/0.68 for cathodic/anodic peaks, respectively, implying that the Li^+^ storage kinetics was both dominated by surface capacitive behaviors and diffusion controlled. Figure 4g presents the electrochemical impedance spectrum (EIS) for further understanding of the Li^+^ diffusion kinetics and electrical resistance of three electrodes at open circuit potential. Generally, Nyquist plots are composed of three compositions: the intersection point on the real axis corresponds to the overall resistance of the cell components (*R*_s_), a semicircle at high frequency associated with the charge transfer resistance (*R*_ct_), and at low frequency line related to Li^+^ diffusion (Z_ω_) [33]. It is apparent that the α/h-MoO_3_ electrode shows smaller diameter and larger slope, suggesting lower charge-transfer resistance and faster Li^+^ diffusion in the electrode, guaranteeing a much better rate performance of the α/h-MoO_3_ electrode. Meanwhile, the Warburg factor (σ) was obtained from the potting of Z′ vs. ω^−1/2^ (Figure 4i). It is noteworthy to mention that the slope of fitted lines of α/h-MoO_3_ electrode is the smaller than that of the other electrodes, suggesting a better Li^+^ diffusion capacity. The above results illustrate that phase engineering is an effective and expandable mean to endow the electrode with fast electrochemical kinetics and a stable structure for superior Li^+^ storage.

## 4. Conclusions

In summary, the α/h-MoO_3_ hetero-phase homojunction was prepared by a partial crystal phase transition. The product exhibited higher rate capability and better cyclic stability. The homojunctions not only showed the enhanced electronic conductivity and Li^+^ diffusion, but also provided more intrinsic active sites and accommodate the volume expansion during discharge/charge. All these simultaneously enhanced the charge storage capability for higher capacity and superior rate performance. Benefiting from these merits, the α/h-MoO_3_ electrode delivered superior electrochemical performance. Specifically, an excellent rate ability (742 mAh g^−1^ at 1.0 A g^−1^) was recorded in this work. Considering the prominent electrochemical performance of α/h-MoO_3_ homojunctions, in situ construction crystal phase homojunctions strategy on polymorph materials provides an alternative solution to the energy storage and related fields.

## Data Availability

Not applicable.

## Supplementary Materials

The following supporting information can be downloaded at: https://www.mdpi.com/article/10.3390/nano12213762/s1, Figure S1: The first CV curves at 0.1 mV s^−1^ of the electrodes. Figure S2: GCD curves at different current densities.
