# Supplementary material for: Storage of Lithium-Ion by Phase Engineered MoO3 Homojunctions
Source: Nanomaterials (Basel). 2022 Oct 26;12(21):3762. doi: 10.3390/nano12213762 (PMC9655550; doi:10.3390/nano12213762)
Supplement: Supplementary file 1 [file nanomaterials-12-03762-s001.zip › nanomaterials-1949664-supplementary.pdf]

Supplementary Materials

For

Storage of Lithium-Ion by Phase Engineered MoO_3 Homojunctions

Dickon H. L. Ng [1,†,*], Sheng Li [2,†], Jun Li [2], Jinning Huang [3], Yingxue Cui [2], Jiabiao Lian [2] and Chuan Wang [3,*]

[1] School of Science and Engineering, The Chinese University of Hong Kong (Shenzhen), Longgang, Shenzhen 518172, China

[2] Key Laboratory of Zhenjiang, Institute for Energy Research, Jiangsu University, Zhenjiang 212013, China

[3] Institute of Advanced Synthesis, School of Chemistry and Molecular Engineering, Jiangsu National Synergetic Innovation Center for Advanced Materials, Nanjing Tech University, 30 Puzhu South Road, Nanjing 211800, China

* Correspondence: dickonng@cuhk.edu.cn (D.N.); ias_cwang@njtech.edu.cn (C.W.)

† These two authors contributed equally to this work.

Supplementary Figures:

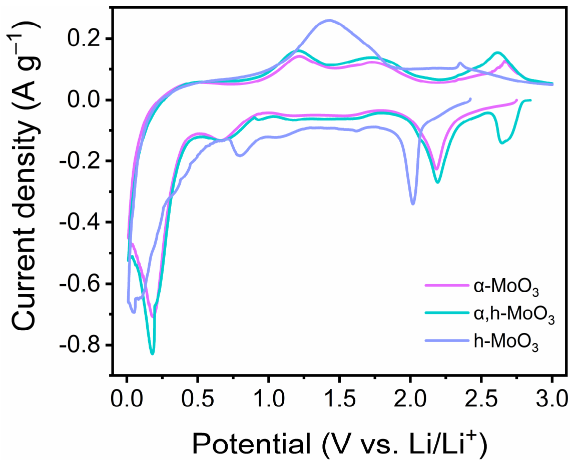

Figure S1. The first CV curves at 0.1 mV s^{-1} of Sample B: α/h-MoO_3; Sample A: h-MoO3; and Sample C: α-MoO3, respectively.

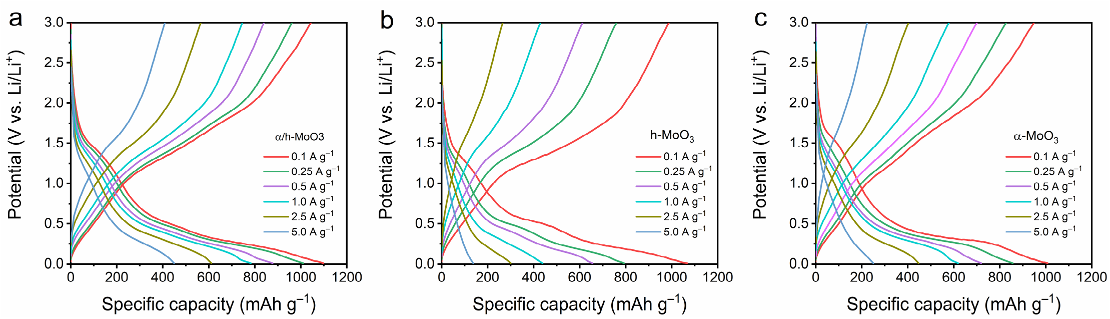

Figure S2. Discharge–charge curves at different current densities of (**a**) α/h-MoO_3, (**b**) h-MoO_3, and (**c**) α-MoO_3.
